# Home-Based Physical Activity Program With Health Coaching for participants With Chronic Obstructive Pulmonary Disease in Sweden: A Proof-of-Concept Pilot Study

**DOI:** 10.1016/j.mayocpiqo.2023.07.005

**Published:** 2023-09-30

**Authors:** Maria V. Benzo, Maria Hagströmer, Malin Nygren-Bonnier, Roberto P. Benzo, Marian E. Papp

**Affiliations:** a Mindful Breathing Laboratory, Mayo Clinic, Rochester, MN; b Division of Family Medicine and Primary Care, Department of Neurobiology, Care Sciences and Society, Karolinska Institute, Sweden; c Division of Physiotherapy, Department of Neurobiology, Care Sciences and Society, Karolinska Institute, Sweden; d Academic Primary Health Care Centre, Region Stockholm, Stockholm, Sweden; e Women’s Health and Allied Health Professionals Theme, Medical unit Occupational Therapy and Physiotherapy, Karolinska University Hospital, Stockholm, Sweden

## Abstract

Home-based interventions are at the center stage of current health care demands. There is a clear need to translate pulmonary rehabilitation into a home-based setting. This 8-week pilot study aimed to determine the feasibility of a home-based physical activity program for participants with chronic obstructive pulmonary disease (COPD) in Sweden. Patients with COPD, aged 40 years or older and clinically stable in the past 3 months, were recruited. The program used a fitness tracker to monitor step count, weekly health coaching calls using motivational interviewing, and video-guided mindful movements. The outcome measures were adherence to the 8-week program’s video-guided exercises (number of times videos were watched), adherence to health coaching calls (minimum 8), monthly and daily step count, and quality of life (QoL) using the chronic respiratory questionnaire. Thirteen participants were enrolled, and 12 participants adhered to health coaching calls and step monitoring. We had 643 video-exercise views, which exceeded the minimum standard (576 views). The mean difference comparing total monthly steps from baseline and the 8-week time point was 47,039 steps (95% CI, –113,625 to 1623.5; *P*=.06). The minimal clinical improvement of 500 daily steps was found for 8 of the patients. No significant improvement was found in the QoL measures and mental health. We found the home-based physical activity program to be a feasible intervention. Patients reported high adherence to tracking step counts, health coaching calls, and video-guided exercise. No improvements in QoL or monthly step count emerged; however, we found high adherence and a positive trend in the number of monthly step counts, and improvements of at least 500 daily step counts improved in most patients with this small sample size.

Chronic obstructive pulmonary disease (COPD) is among the most common chronic respiratory diseases 65 million individuals have the disease. Globally COPD is suggested to be the third leading cause of death in 2030. Chronic obstructive pulmonary disease is a leading cause of death and disability in high, medium, and low-income countries, and using pulmonary rehabilitation as an integral, non-pharmacological component is important in disease management. It is a progressive lung disease that makes breathing difficult, usually worsens over time, and eventually may make it difficult for people to carry out regular, simple daily activities.

Pulmonary rehabilitation (PR) is an intervention that combines the promotion of regular physical activity and self-management^1^, ^2^, ^3^ to maintain health and functionality in patients with COPD.^4^ It is regarded as an essential component of care for people with COPD and is supported by strong scientific evidence.^5^ Among patients with COPD, a sedentary lifestyle considerably increases mortality and is an important risk factor for exacerbation of COPD.^6^

Despite the proven efficacy of PR, its adoption remains low. Only 20% of eligible outpatients and 2% of COPD patients posthospitalization attend center-based PR.^7^ In Sweden, only 36% of patients with COPD report having sufficient physical activity levels (30 minutes per day and 5-7 days a week). Most of primary care health clinics (92%) have a physical and respiratory therapist, but few (25%) report having access to PR.^8^

Transportation, accessibility, and physical and emotional frailty are the main barriers to adoption and adherence to the current center-based PR.^9^^,^^10^ A home-based approach can potentially offer health benefits without the aggregated risk of exposure. Health coaching is increasingly used in home-based lifestyle programs^11^ and has reported effectiveness in facilitating patient improvement in various aspects of health by affecting patients’ knowledge, skills, self-efficacy, and behavior change.^11^, ^12^, ^13^, ^14^, ^15^

Health coaching may use motivational interviewing (MI) and goal setting to empower patients to take an active role in managing their health conditions and making important health behavior changes.

This novel home-based physical activity (HPA) program is on the basis of integrative movement techniques and health coaching and allows participants with COPD to perform physical activity at home. Our goal was to determine the feasibility and adherence of the HPA program with patients with COPD in Sweden and determine its effect on the number of views of video-guided exercises, step count, and disease specific QoL.

## Methods

### Study Design

We performed an 8-week pilot study that evaluated both process and scientific feasibility to a novel HPA program with participants with COPD in Sweden. On the basis of an earlier feasibility study in the United States a sample size of 10+ is sufficient to evaluate the easibility of implementing an intervention program. This pilot study preceded the future randomized control trial, Trial registration: Clinical trials NCT04820257, date 29/03/2021.

### Selection and Enrollment of Participants

Participants diagnosed with COPD receiving outpatient care were recruited at university hospitals, health care units, websites, and lung facilities in Stockholm and Sweden. Written and oral informed consent was collected from all participants before enrollment.

### Inclusion Criteria

All genders, age 40 years and older; forced expiratory volume in one second (FEV1) <80% as documented by pulmonary function; medical diagnosis of COPD; access to a smartphone or computer tablet with an internet connection.

### Exclusion Criteria

Patients were excluded if they were unable to perform low-intensity exercise; inability to follow commands (participants with disorientation or severe neurologic or psychiatric conditions).

### Intervention

Participants were instructed to complete a daily video-guided movement practice (VGM) (tai chi and yoga) lasting from 6 to 60 minutes. Participants were encouraged to engage in the VGM 6 days per week, for a total of 48 days (8 weeks). The VGM included coordinated movements with breaths to benefit the strength and flexibility of the upper extremities and thorax with mindful walking. The VGM were standing and sitting poses and breathing exercises with an emphasis on extended exhalation, props were used as chairs and blocks.

The full version of the VGM is available online.^16^ Daily step counts were measured using a commercially available wrist activity monitor (Garmin vivofit 4, Garmin, Schaffhausen, Switzerland).^17^ The activity monitor was synced to the patient’s smartphone through the Garmin connect app. The health coach reviewed the daily step data and discussed this information with the participant in the health coaching call.

The participants received a scheduled weekly health coaching call lasting 10-30 minutes during the 8 weeks. The health coaching sessions were designed to increase awareness of the physical activities completed and ignite intrinsic motivation for healthy behaviors. Collaboratively, exercise, and step goals were set. Health coaches had access to patient’s daily and weekly step counts to aid the coaching discussion and goal setting. The participant interacted with the same health coach throughout the whole program.

The calls were structured using MI and followed the protocol published by Benzo et. Al.^14^ Health coaches from different professions (physiotherapists, MD, personal trainers, and behavioral specialists) were trained by the mindful breathing laboratory in basic MI skills in health coaching by virtual zoom lectures, they also had MI skills from other courses.

### Potential Risks

Risk associated with completing the daily routine was minimal to low, with the exercises being of low intensity.^13^

### Outcomes

We considered the feasibility measures to be the adherence rate, defined by the completion of 8 weeks of health coaching, the dropout rate, the use of the fitness tracker, and the adherence to the VGM. Demographic characteristics and patient characteristic information was retrieved at baseline. Measures of health used was the visual analog scale, was used ranging from 0-100, a higher number indicated better health. The chronic respiratory disease questionnaire self-administered standardized was used to measure QoL^18^ measuring 20 items across 4 dimensions (dyspnea, fatigue, emotion, and mastery).

The total daily, weekly, and monthly steps were collected from the fitness tracker website. The data was imported, stored, encrypted and deidentified into a secure computer. The device was synced by bluetooth to an app (Garmin connect) on the participant's smartphone. An account was created with deidentified data using the participant's study ID and prevented personal information from being disclosed for others than the health coaches and participants to review. The paper questionnaires were mailed to the participants and were handled and deidentified by the principal investigator (M.P.) for data entry. The health coaching calls were not recorded.

### Statistics

The difference between total monthly steps for the 2-time points (first month and the second month) was not normally distributed. Therefore, the nonparametric Wilcoxon test was used. The chronic respiratory disease questionnaire scores were normally distributed, thus, a paired *t* test was used to see if there is a difference between time points, R Core Team (2021). The Shapiro-Wilk method was used to test for normality. All analyses were performed using STATA 14 (Stata Corp) and R Core Team (2021).

## Results

Thirteen eligible participants were enrolled through queries from September 2021 to December 2021, with one dropout. 12 participants completed 8 weeks of health coaching calls (minimum 8) and used the fitness tracker. The adherence with the VGM was determined by the number of times the videos were watched on the online platform (KI play). On the basis of our recommendation, we expected to have at least 576 views. Our data shows a total of 643 views (excluding pursed lip breathing, which had 80 views) exceeding our minimum standard. The views for VGM were 316 for seated and standing flexibility movements, 187 for balance practice, and 140 for yoga-based movements during the 8-week intervention.

No significant changes from baseline to follow-up in QoL emerged (Table 1).

**Table 1:** Baseline and 8-Week Parameters in COPD Participants Performing Home-Based Pulmonary Rehabilitation (n=12)

Parameter	Baseline	8 week
Sex F/M	8/4	—
Age (y), median (range)	70.5 (55-83)	—
Perceived health very high/high/moderate/low/very low	0/7/2/3	2/6/2/2
Self-rated health (VAS) mean; median (range) (100=maximal health)	66.8; 68.5 (45-91)	65.2; 71 (40-85)
CAT mean; median (range)	14.7;15 (5-24)	13.2; 12.5 (5-24)

Abbreviations: CAT, COPD assessment test; COPD, chronic obstructive pulmonary disease; F, female; M, male.

Total monthly steps were analyzed, such as the average steps during the first 4-weeks of intervention were compared with the second 4-week period (Table 2). Individual average weekly step counts for each participant are plotted in Figure. We found an upward trend in steps over the period, although not statistically significant. Eight of 12 participants improved at least 500 daily steps after 8 weeks.

**Table 2:** Average Monthly Step Count in Participants (n=12) with COPD^a^

	Month 1 average (SD)	Month 2 average (SD)	Average difference^b^	95% CI	*P*
Average monthly steps	136,348 (80,429)	183,387 (108,983)	47,039	(−113,625 to 1623.5)	.06

a Abbreviations: COPD, chronic obstructive pulmonary disease; SD, standard deviation.

b Average differences were calculated from 8-week scores minus baseline scores.

**Figure:**
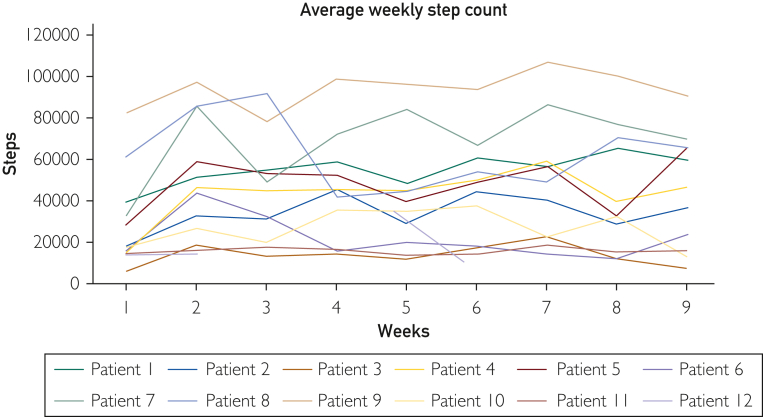
Average weekly step count and trend for each COPD participant. COPD, chronic obstructive pulmonary disease.

## Discussion

In this study, we evaluated the feasibility of a novel HPA for patients with COPD in Sweden. The intervention was feasible with respect to adherence to the health coaching calls, tracking steps, and the video-guided exercise. The HPA had a low dropout rate. A positive trend in the monthly step count was found.

Aligning with current publications, we believe that remote monitoring by the health coach and self-monitoring may influence participants to be more physically active.^11^ Regular feedback on a participant’s daily step count is central to the self-awareness that leads to behavior change and appropriate coaching.^6^^,^^19^^,^^20^ No significant changes in QoL, step counts, self-rated health emerged and could be related to power. Others report with larger samples, improvement and maintenance of daily step counts in COPD.^21^ The difference between the total steps in the first month compared with the second month found an upward trend but did not reach statistical significance. However, not significant, we observed the recommended minimal clinical improvement in steps in 8 of 12 participants on the basis of a 500 steps cutoff.^22^^,^^23^ Larger studies found a significant increase in the daily steps of COPD participants with the use of a pedometer together with health coaching, using individualized goals and personalized motivational messages.^24^

Swedish primary health care struggles with waiting lists for health consultations and unequal care.^25^ Telemedicine services can increase the potential choices for patients. Swedish treatment guidelines encourage self-management and education through PR; however, uptake and adherence are limited.^26^ The urgent need to find new methods to facilitate the provision of self-management support to patients with COPD leads to the innovation of telehealth solutions. Telehealth and electronic health solutions are a promising way of delivering health services, such as self-management education, physical activity programs, health coaching, and PR, to patients with COPD.^12^ In alignment with this pilot, allowing access to telehealth solutions in addition to usual care may be an effective strategy to promote self-management and physical activity. In Sweden, telehealth is perceived as efficient and safe, according to users.^25^

In addition, it provides an alternative care service that saves time and resources for patients, the health care system, and the environment. This pilot study was created in the context of a Karolinska Institute and Mayo Clinic research collaboration. We had based our home-based intervention on the mindful breathing laboratory at Mayo Clinic exercise videos that are currently and previously used in their trials.^27^ A more comprehensive home-based PR program recently published^11^ has shown effective improvements in QoL in patients with COPD. Other comprehensive studies support our hypothesis that low-intensity exercise can improve health outcomes in COPD. The Copenhagen City Heart Study found that patients reporting low levels of exercise (light physical activity for 2 hours a week), compared with very low levels of exercise (none) reported a significantly lower risk of COPD admissions and mortality.^28^ In the context of the COVID-19 pandemic, a home-based program, like the one tested in this pilot, may alleviate the accessibility barrier of center-based PR and allow these vulnerable patients to gain the benefit of practicing regular physical activity without an additional risk of exposure to COVID-19 infection.

### Strengths and Limitations

The study has several limitations that should be acknowledged. The health care providers were collaborators and their dedication to health coaching depended on their work schedule, which could have limited the intervention delivery.

Most of the coaches had valid MI training; however, some of the coaches were not certified. Coaches were invited to lectures, workshops, and provided reading material by the mindful breathing laboratory at Mayo Clinic, but no evaluation was determined, and no fidelity process of the health coaching was done.

The season was commonly reported as a barrier by participants, the colder weather limited them from being more active outside of their homes. Our sample did not have mental issues as general anxiety (low general anxiety disorder score) and any large changes were not expected owing to a floor effect.

Regarding the exercise videos, they had the audio in English and subtitles in Swedish, which created a language barrier for some participants and difficulties to read while doing the movements. This might have been a hindering factor that made their decision not to continue the practice and preferred to do their exercises mainly by walking outside. Further research should include exercise videos in Swedish to create even higher engagement, and this is planned in an upcoming randomized controlled trial.

We suggest continuing this line of research with the home-based approach in future trials with a larger sample.

## Conclusion

This HPA program was found to be a feasible intervention. Patients reported high adherence to coaching calls, video-guided exercise, and tracking of step counts. However, since pilot design, no improvements in the QoL and monthly step counts emerged. However, we found a positive trend in monthly step counts. This study might provide valuable information, as we have described in the limitations for further research and implementation of home-based programs.

## Potential Competing Interests

All authors have no conflict of interest.
